# A vertebrate-conserved *cis*-regulatory module for targeted expression in the main hypothalamic regulatory region for the stress response

**DOI:** 10.1186/s12861-014-0041-x

**Published:** 2014-11-27

**Authors:** Jose Arturo Gutierrez-Triana, Ulrich Herget, Patrick Lichtner, Luis A Castillo-Ramírez, Soojin Ryu

**Affiliations:** Developmental Genetics of the Nervous System, Max Planck Institute for Medical Research, Jahnstrasse 29, D-69120 Heidelberg, Germany; Current address: Centre for Organismal Studies (COS), University of Heidelberg, Im Neuenheimer Feld 230, D-69120 Heidelberg, Germany; The Hartmut Hoffmann-Berling International Graduate School of Molecular and Cellular Biology, University of Heidelberg, Heidelberg, Germany

**Keywords:** Otp, Evolutionarily conserved non-coding regions, *cis*-regulatory module, Hypothalamic-pituitary-adrenal axis, Neurosecretory preoptic area, Cortisol, Zebrafish, Stress response

## Abstract

**Background:**

The homeodomain transcription factor orthopedia (Otp) is an evolutionarily conserved regulator of neuronal fates. In vertebrates, Otp is necessary for the proper development of different regions of the brain and is required in the diencephalon to specify several hypothalamic cell types, including the cells that control the stress response. To understand how this widely expressed transcription factor accomplishes hypothalamus-specific functions, we performed a comprehensive screening of *otp cis*-regulatory regions in zebrafish.

**Results:**

Here, we report the identification of an evolutionarily conserved vertebrate enhancer module with activity in a restricted area of the forebrain, which includes the region of the hypothalamus that controls the stress response. This region includes neurosecretory cells producing Corticotropin-releasing hormone (Crh), Oxytocin (Oxt) and Arginine vasopressin (Avp), which are key components of the stress axis. Lastly, expression of the bacterial nitroreductase gene under this specific enhancer allowed pharmacological attenuation of the stress response in zebrafish larvae.

**Conclusion:**

Vertebrates share many cellular and molecular components of the stress response and our work identified a striking conservation at the *cis*-regulatory level of a key hypothalamic developmental gene. In addition, this enhancer provides a useful tool to manipulate and visualize stress-regulatory hypothalamic cells *in vivo* with the long-term goal of understanding the ontogeny of the stress axis in vertebrates.

**Electronic supplementary material:**

The online version of this article (doi:10.1186/s12861-014-0041-x) contains supplementary material, which is available to authorized users.

## Background

The homeodomain protein Orthopedia (Otp) belongs to an ancient family of transcription factors involved in cell fate specification in the central nervous system of invertebrates and vertebrates [1-3].

In vertebrates, Otp is required for the proper development of the neuroendocrine hypothalamus. Genetic analyses in mice, and more recently in zebrafish, have shown that Otp, together with the heterodimeric complex formed by the bHLH-PAS transcription factors Sim1 and Arnt2, is required for the development of virtually all neuroendocrine cells in the hypothalamus. When Otp and Sim1 functions are eliminated due to a genetic mutation or morpholino injection, the cells expressing Arginine vasopressin (Avp), Oxytocin (Oxt), Corticotropin-releasing hormone (Crh), Thyrotropin-releasing hormone (Trh) or Somatostatin (Ss) fail to develop [3-11]. Interestingly, in the protostome polychaete *Platynereis dumerilli*, Otp is expressed in the medial neurosecretory forebrain region and overlaps with vasotocinergic and RFamidergic neurons [12]. These results indicate an evolutionarily conserved role of Otp for the specification of the neurosecretory control centers in bilaterian animals [13].

The hypothalamic neurosecretory control center is a component of the hypothalamic-pituitary-adrenal or hypothalamic-pituitary-interrenal axis (HPA axis or HPI axis in fish), which carry out, among other functions, a series of integrated and hierarchical physiological responses mediating the interaction of an animal with the environment under stress [14]. This system is considered to be a vertebrate innovation, such that many cellular and molecular components are found in cyclostomes and gnathostomes but not in cephalochordates and urochordates [15-17]. Exogenous and endogenous environmental factors can activate the HPA axis and stimulate specific neurosecretory cells in the hypothalamus to secrete hormones such as Crh, Avp and Oxt. These hormones reach the pituitary and trigger the release of adrenocorticotropic hormone (ACTH), which then stimulates the production of glucocorticoids (GCs) in the adrenal or interrenal gland [18,19]. Downstream effects are then mediated by the action of GCs in peripheral organs as well as their central targets [20,21].

Although the most well-known function of Otp is within the neuroendocrine hypothalamus, Otp is also expressed and involved in the specification of different cell types in multiple regions of the brain [22,23]. For example, just within the hypothalamus, Otp expression can be found in the retrochiasmatic, ventral tuberal and mammillary areas in the mouse and in the posterior hypothalamus in the zebrafish [2,23,24]. Furthermore, in the posterior hypothalamus of the zebrafish, Otp plays a crucial role in the specification of vasoactive intestinal peptide (VIP)-producing cells [23]. Its broad expression and involvement in the specification of different cell types raises the question as to how Otp achieves regional and cell-type specific expression and functions. In particular, we asked how Otp expression and function in the neuroendocrine hypothalamus is mediated in contrast to other regions. To address this question, we chose to analyze the regulatory landscape influencing the spatial expression of the *otp* gene. Specifically, we used zebrafish as a model system to identify *cis*-regulatory modules that drive region-specific expression of the *otp* gene particularly in the neuroendocrine hypothalamus that controls the stress response. The zebrafish is a very tractable organism for experimental regulatory genomics due to the availability of transgenic tools that allow visualization of fluorescent proteins reporting the regulatory potential of discrete genomic elements in a transparent embryo [25,26]. Further, the zebrafish is a promising model organism for stress research [27]. The hypothalamic region that controls the stress response is found in the paraventricular nucleus (PVN) in mammals. Its homologous region is found in the neurosecretory preoptic area (NPO) in the larval zebrafish [28]. While previous studies have analyzed *cis*-regulatory regions of the *otp* gene in zebrafish, none of the identified regions were sufficient to restrict *otp* expression to the NPO [29,30].

Vertebrate genomes share highly conserved non-coding sequences mainly clustered close to genes involved in embryonic development and likely involved in the genomic circuitry of these genes [31]. Therefore, in the present work, we screened highly conserved non-coding regions of the *Otp* gene of vertebrates and identified a regulatory element with activity in a restricted area of the forebrain. Among the cells labeled by this enhancer, we found the neurosecretory cells of the HPI axis that produce Crh, Oxt or Avp but not the diencephalic dopaminergic (DA) cells. When combined with a genetic ablation technique, this regulatory element allowed attenuation of the stress response. Taken together, these data suggest that a modular evolutionarily constrained regulatory code has been used by the gene *Otp* in gnathostomes to specify cells in the neurosecretory hypothalamus.

## Results

### The vertebrate Otp gene is flanked by evolutionarily conserved non-coding sequences

To identify and characterize functional *cis*-regulatory elements of the *otp* gene, we used a phylogenetic footprinting approach [25,32] to screen for evolutionarily conserved non-coding sequences (ECRs) in or around the gene using the ECR browser (http://ecrbrowser.dcode.org) and compared the genomic sequences flanking the *otp* gene of human, mouse and zebrafish (*otpa*). To determine whether these ECRs are conserved in different vertebrates, we performed individual pairwise BLASTn comparisons using the standard nucleotide Blast [33] optimized for somewhat similar sequences using the genome databases of the National Library of Medicine (http://blast.ncbi.nlm.nih.gov), Ensembl 2014 (http://www.ensembl.org [34]) and the available genome sequences of the sarcopterygian Coelacanth (*Latimeria chalumnae*), the chondrichthyan elephant shark (*Challorhincus milii*), and agnathan lampreys (*Lethenteron japonicum* and *Petromyzon marinus*) (http://www.ensembl.org/Latimeria_chalumnae; http://esharkgenome.imcb.a-star.edu.sg [35]; http://jlampreygenome.imcb.a-star.edu.sg [36]). The minimum length of the regions was set to 80 bp, and the identity was set to above 70%.

**Figure 1:**
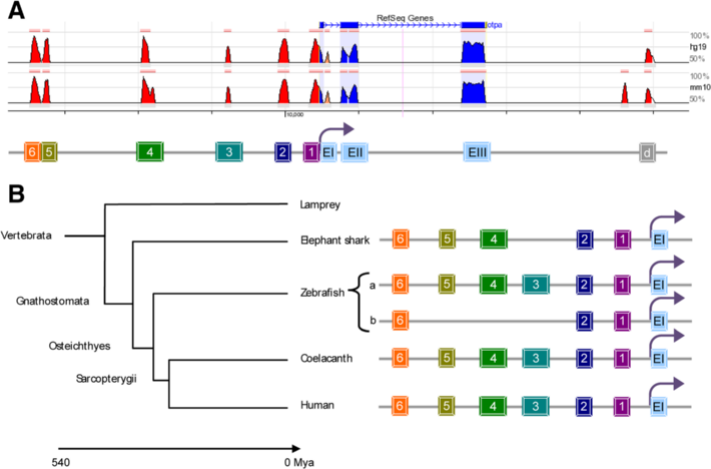
**Genomic structure and evolutionarily conserved non-coding regions (ECRs) of the** ***otp*** **gene. A**, Graphical comparison of the *otp* locus, according to the ECR browser, showing intergenic ECRs (red) and exons (blue) in the zebrafish (genome assembly danRer7), the human (genome assembly hg19) and the mouse (genome assembly mm10). We used the sequence of the *otpa* gene from zebrafish as the reference. **B**, A schematic phylogenetic tree of vertebrates. *otp* ECRs are evident only in gnathostome vertebrates. We noted that some ECRs were absent in the paralogous gene *otpb* in zebrafish (see text for more details).

Several ECRs with more than 70% identity were identified, including 6 ECRs in the 5′ upstream region and 1 in the 3′ downstream region (Figure 1A). Interestingly, all of the 5′ upstream ECRs, except for *otp*ECR3, are present in the upstream region of the *otp* gene in gnathostome vertebrates. The overall percentage of identity is remarkably high (>92% for *otp*ECR5, >85% for *otp*ECR4, >83% for *otp*ECR2, >80% for *otp*ECR6, >78% for *otp*ECR1, and >72% for *otp*ECR3, see Additional file 1: Figure S1). In the pairwise comparisons of human and zebrafish, the values are similar to the global comparison (92% for *otp*ECR5, 88% for *otp*ECR4 and *otp*ECR2, 86% for *otp*ECR6, and 78% for *otp*ECR3 and *otp*ECR1). Interestingly, in the zebrafish paralogous *otp* gene, *otpb*, ECR3, ECR4, and ECR5 are absent. Furthermore, the percentage of identity of the remaining *otpb* ECRs is lower than the identity of the orthologous blocks in the vertebrates analyzed (e.g., >59% for *otp*ECR6). Although an *otp* gene is found in the genome of the lamprey, none of the ECRs were identified under the parameters used for the analysis (Figure 1B), suggesting an innovation in the regulatory network controlling this gene in jawed vertebrates.

### *otp*ECR6 is a *cis*-regulatory module driving expression in the NPO of the zebrafish brain

**Figure 2:**
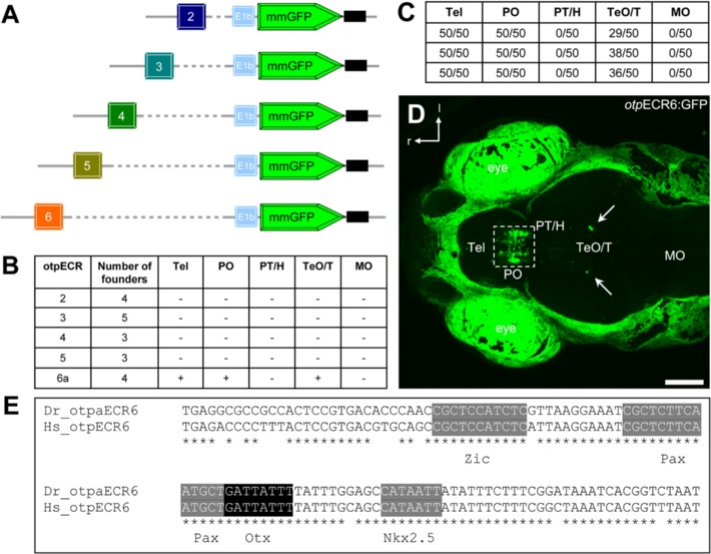
**Enhancer activity displayed by stable** ***otp*** **ECR-transgenic lines. A**, A schematic depiction of constructs used to generate the transgenic lines. **B**, Enhancer activity displayed by transgenic lines analyzed at 3-5 dpf. The table shows the GFP levels and expression patterns observed in cardiac-RFP positive founders for the individual *otp*ECRs. **C**, Numbers of larvae showing GFP+ cells within different brain regions (Data from 3 independent clutches are shown, n = 50 per clutch). In all animals, the caudal telecephalic-preoptic cluster is present, and in many animals, few ectopic cells appear in the tectal-tegmental region. **D**, Confocal z-stack maximum projection of a dorsal *in vivo* view of *Tg(otpECR6-E1b:mmGFP)* expression. GFP is expressed in a dense cluster within the preoptic area and extends into the caudal telencephalon. In many larvae, a few isolated ectopic cells can be detected in the midbrain. **E**, The evolutionarily conserved region *otp*ECR6 contains predicted binding sites for transcription factors belonging to the families Otx, Pax, Nkx and Zic. The *otp*ECR6 DNA sequences of zebrafish (Dr) and human (Hs) were aligned and analyzed using the multiF program (https://multitf.dcode.org of the ECR browser). Abbreviations: Tel, telencephalon; PO, preoptic area; PT, posterior tuberculum; H, hypothalamus; TeO, optic tectum; T, tegmentum; MO, medulla oblongata; r, rostral; l, lateral. Scale bar: 100 μm.

To evaluate the regulatory activity of the ECRs identified in the vertebrate *Otp* genes, we analyzed the expression of individual ECRs in transgenic zebrafish using GFP as a reporter. To do this, individual ECRs around the *otpa* gene, from 2 to 6, were cloned into a modified version of the ZED vector (a Tol2-based plasmid carrying insulator sequences to decrease variability in the GFP expression pattern due to integration site effects [37]). The plasmids also contained a cassette expressing RFP under a cardiac promoter as a transgenesis marker. These plasmids were injected into zebrafish embryos and analyzed at 24 hours post-fertilization (hpf), 72 hpf, and 5 days post-fertilization (dpf) in both transient assays and using stable transgenic fish lines. At least three independent stable transgenic lines were established per plasmid. In our analyses, we excluded ECR1, which contains the TATA box, because this region performed as a basal promoter that could be used to trap neighboring enhancers. For instance, from our initial study, one of the transgenic zebrafish lines using the *otp*ECR1 showed GFP expression specifically in the habenula, although *otpa* is normally not expressed there [38]. Notably, we failed to detect GFP expression in all ECRs except *otp*ECR6 in transgenic lines that were identified using the RFP expression in the heart (Figure 2B). *otp*ECR6 drives a robust forebrain expression even in transient-injected animals (data not shown). Further characterization was performed using the stable line *Tg(otpECR6-E1b:mmGFP)*. In anatomical terms, this region corresponds primarily to the zebrafish neurosecretory preoptic area (NPO) and the dorso-rostral extension of the *otpa* domain in the caudal telencephalon [28]. We also observed ectopic and mosaic GFP expression in a small group of cells in a dorsal region of the posterior midbrain of larvae (Figure 2C,D). This mosaicism probably reflects multiple insertions of the transgene in the founder genome, as is reported for Tol2-mediated transgenesis in zebrafish [39].

The conserved DNA sequence of *otp*ECR6 contains predicted binding sites for transcription factors belonging to the families Otx, Pax, Nkx and Zic (Figure 2E). To test for regulatory interactions of members of any of these TF families with the *otp*ECR6 module, we co-injected, into one-cell stage embryos, the reporter plasmid *otp*ECR6-E1b:mmGFP, which contains *otp*ECR6 fused to the E1b basal promoter and the fluorophore mmGFP, together with mRNA encoding a chimeric protein consisting of a candidate transcription factor fused to the strong activation domain of the VP16 protein [40]. We then checked for GFP expression at 6-10 hours post fertilization (hpf). This transcriptional activation assay was performed using the zebrafish transcription factors Otx1, Otx2a, Pax2a, Pax6a, Nkx2.1a, Nkx2.1b and Zic2a, which are expressed in the hypothalamus (www.zfin.org). None of these transcription factors activated the expression of GFP (data not shown).

**Figure 3:**
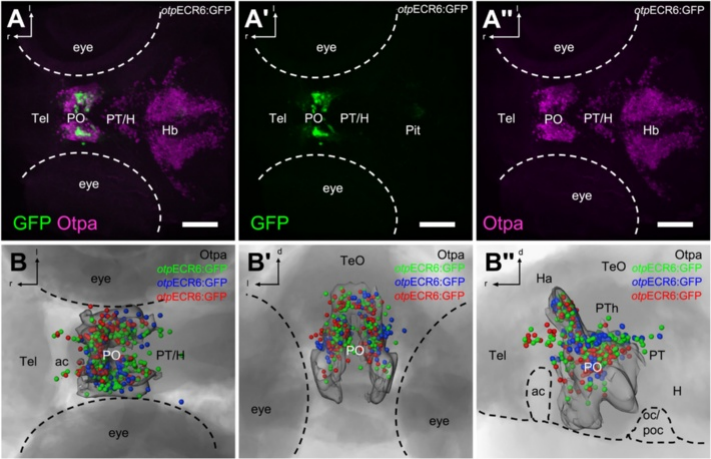
***otp*** **ECR6 displays enhancer activity in the forebrain with expression in cells localizing within the caudal telencephalon and dorsal half of the preoptic Otpa domain.** **A**, Confocal z-stack maximum projection of an immunohistochemically stained 5 dpf *Tg(otpECR6-E1b:mmGFP)* larva double-labeled for GFP (A’) and Otpa (A”). **B**, Reconstructed localization and variability of GFP-expressing cells. Colors represent three representative maps derived from three different animals after registration using the outlines of the Otpa signal as references (transparent surface). Abbreviations: Tel, telencephalon; PO, preoptic area; PT, posterior tuberculum; H, hypothalamus; Hb, hindbrain; Pit, pituitary; ac, anterior commissure; TeO, optic tectum; Ha, habenula; PTh, prethalamus; oc, optic chiasm; poc, postoptic commissure; r, rostral; l, lateral; d, dorsal. Scale bars: 100 μm.

In the *Tg(otpECR6-E1b:mmGFP)* line, GFP-expressing cells appear starting at approximately 28-30 hpf and are mostly found in the forebrain. At 5 dpf, 91% of the GFP cells are *otpa*-positive, as shown by immunostaining (Figure 3). Therefore, we conclude that *otp*ECR6 is a *cis*-regulatory element driving modular expression mainly in a subset of *otpa*-expressing cells in the forebrain of zebrafish.

### Chemoarchitecture defined by *otp*ECR6

**Figure 4:**
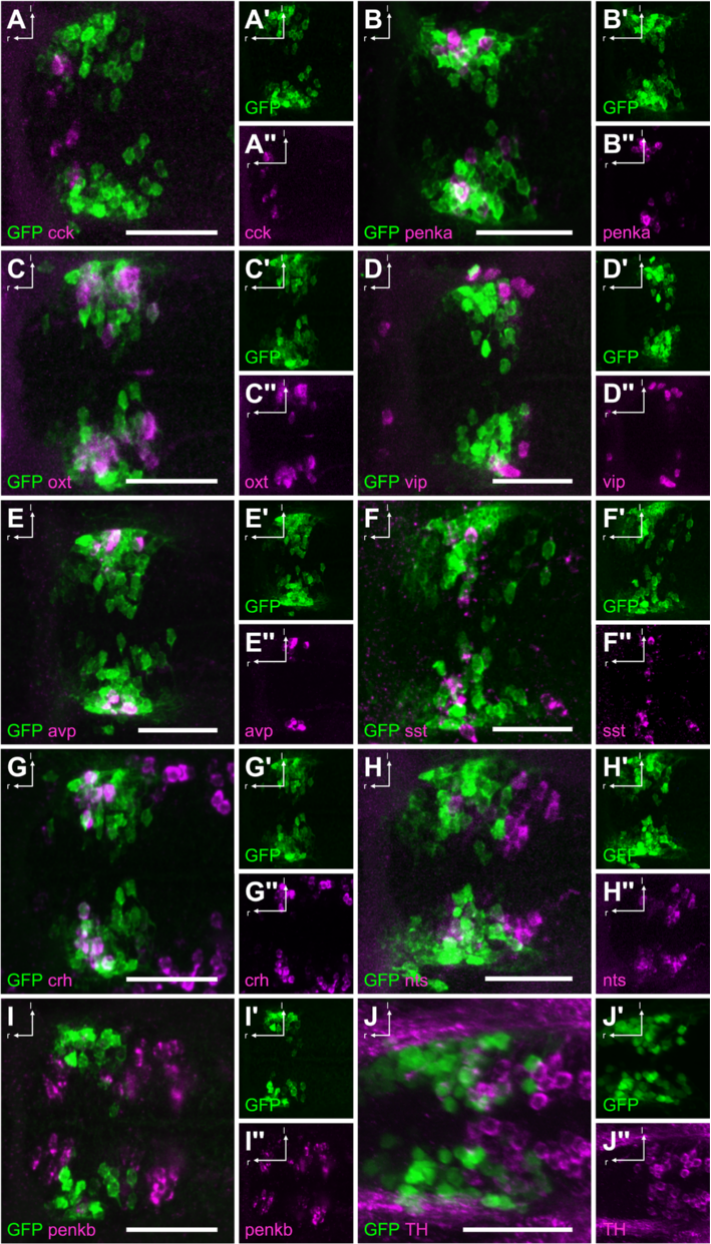
**NPO cells involved in the HPA axis are labeled in the** ***Tg(otpECR6-E1b:mmGFP)*** **transgenic line. A**, There is no overlap of GFP with *in situ* stained *cck+* cells. **B**, There is some overlap of GFP with *penka+* cells. **C**, There is extensive overlap of GFP with *oxt+* cells. **D**, There is no overlap of GFP with *vip+* cells. **E**, There is a high degree of overlap of GFP with *avp+* cells. **F**, There is some overlap of GFP with *sst1.1+* cells. **G**, There is a high degree of overlap of GFP with *crh +* cells. **H**, There is some overlap of GFP with *nts+* cells. **I**, There is no overlap of GFP with *penkb +* cells. **J**, There is no overlap of GFP with immunostained TH+ cells. Abbreviations: r, rostral; l, lateral. Scale bars: 50 μm.

**Figure 5:**
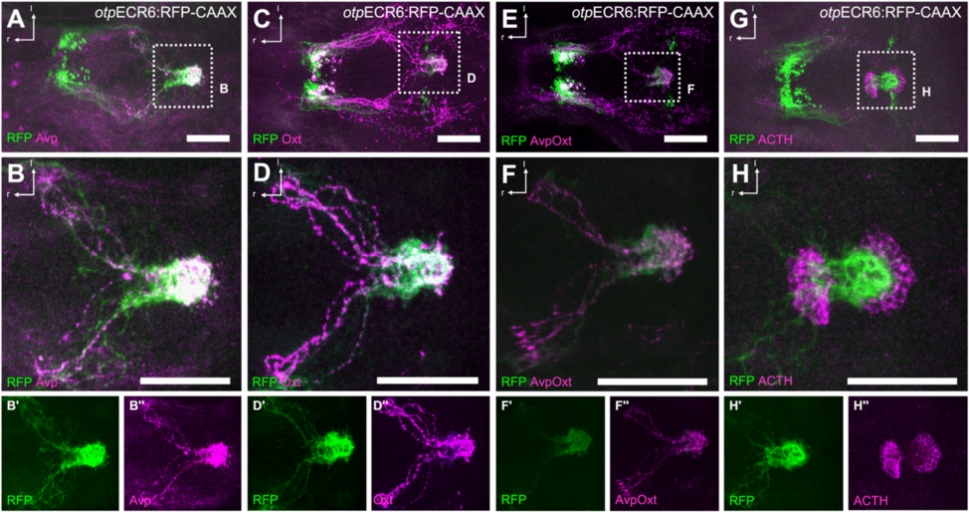
**A group of cells labeled by the activity of the** ***otp*** **ECR6 enhancer project to the pituitary. A-D**, Costaining of RFP with Avp **(A)** or Oxt **(C)** as typical hypophysiotropic cell types shows a high degree of overlap of fibers reaching the pituitary (magnified views in **B** and **D**). **E**, Costaining of RFP with Avp and Oxt combined (magnified view in **F**). **G**, Costaining with ACTH as a pituitary marker confirms the dense RFP bundles as part of the pituitary (magnified view in **H**). Abbreviations: r, rostral; l, lateral. Scale bars: 100 μm.

To characterize the cell identity of the GFP-expressing cells driven by *otp*ECR6, we performed immunohistochemistry and fluorescent *in situ* hybridization (FISH) using probes and antibodies against different neuropeptidergic markers previously described to be present in this region [28]. GFP cells in the NPO colocalized with neurosecretory cells expressing *crh, avp, oxt,* or *proenkephalin a (penka)* (Figure 4). 89% of the *oxt*–positive cells, and 81% of the *avp*–positive cells (based on IHC) showed colocalization with GFP; while 62% of the *crh*-positive cells, and 22% of the *penka*–positive cells (based on FISH) showed colocalization with GFP. In contrast, diencephalic DA cells and their projections were not included in the GFP expression domain (Figure 4). There was no colocalization with cells producing *cholecystokinin (cck)*, *vip*, or *proenkephalin b (penkb)*, and low colocalization with cells producing *somatostatin (sst1.1)* or *neurotensin (nts)*. Because *cck*, *vip*, *penkb*, and *nts* cluster at the border of the NPO, we concluded that *otp*ECR6 expression overlaps more with the central core region of the NPO, where *crh*, *avp*, *oxt*, and *penka* are clustered. A morphological hallmark of hypothalamic neurosecretory cells is their projection to the pituitary. To confirm the neurosecretory nature of the cells targeted using *otp*ECR6, we generated a transgenic line expressing a membrane-tagged RFP to visualize cell projections. Indeed, we observed two sets of bilateral projections in *Tg(otpECR6-E1b:RFP-CAAX).* One set projects caudally, ending halfway along the spinal cord (not shown), whereas the other projects to the pituitary, as shown by co-staining with an ACTH antibody (Figure 5). The RFP projections toward the pituitary colocalize with Oxt and Avp projections. Taken together, our results indicate that *otp*ECR6 is a functional regulatory element with activity in key neurosecretory cells of the NPO.

### Genetic cell ablation using *otp*ECR6 impairs the stress response

**Figure 6:**
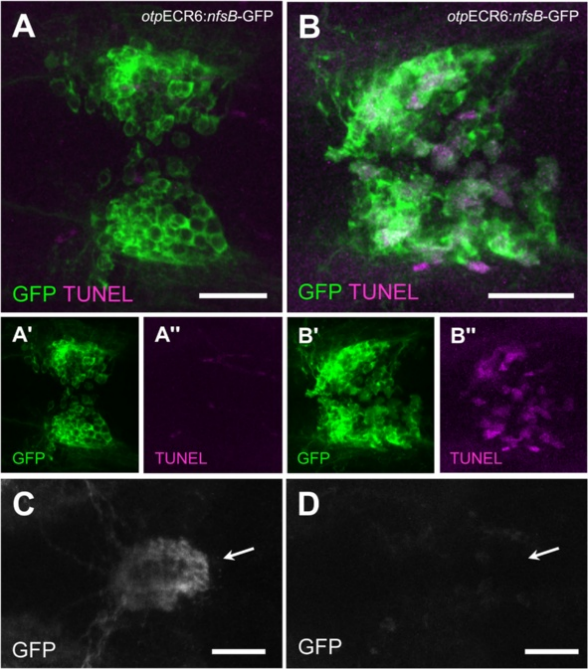
**Nitroreductase-expressing cells in** ***Tg(otpECR6-E1b:nfsb-GFP)*** **larvae display a reduction in the number of projections to the pituitary and an increase in apoptotic bodies when exposed to Mtz. A**, TUNEL staining of the preoptic GFP expression domain in an untreated larva. **B**, TUNEL staining of the preoptic GFP expression domain in an Mtz-treated larva. **C**, Pituitary bundles of GFP-stained fibers in a control larva (arrow). **D**, The pituitary location in an Mtz-treated larva (arrow). Note that in the control larva, the GFP fibers reach the pituitary and no apoptosis is detected, but in the Mtz-treated larva, the pituitary innervation is disrupted and apoptosis is apparent in preoptic cell bodies. Scale bars: 50 μm.

The conditional nitroreductase-metronidazole (NM) system has been recently used in zebrafish to determine the function of a specific group of cells in a developing organism [41,42]. Because the *otp*ECR6 regulatory element drives expression in neurosecretory cells involved in the HPI axis response, we hypothesized that expressing the nitroreductase protein under this regulatory element will impair the stress response when the larvae are incubated in metronidazole (Mtz). To test this directly, we generated stable transgenic zebrafish lines expressing the *E. coli* nitroreductase *nfsB* as a GFP fusion protein under the *otp*ECR6. To test the efficiency of the NM method, we tested the ablation efficiency of *nfsB-*GFP cells in *Tg(otpECR6-E1b:nfsb-GFP)* larvae exposed to Mtz using a TUNEL assay. At 6 dpf, we detected TUNEL-positive cells in the NPO area only in those *Tg(otpECR6-E1b:nfsb-GFP)* larvae treated with Mtz (Figure 6A, B). In addition, we observed a significant decrease in the number of projections to the pituitary in Mtz-treated embryos compared with untreated embryos (Figure 6C, D).

**Figure 7:**
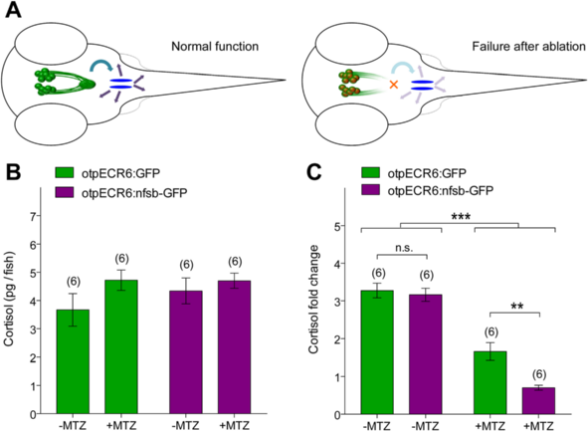
**Mtz-induced impairment of the cortisol response to stress in** ***Tg(otpECR6-E1b:nfsb-GFP)*** **larvae. A**, A schematic illustration of a functional stress axis (fibers of the hypothalamo-pituitary complex in green, interrenal gland in blue) and an impaired stress axis after Mtz treatment. **B-C**, Basal and stress-induced cortisol levels in *Tg(otpECR6-E1b:mmGFP)* and *Tg(otpECR6-E1b:nfsb-GFP)* larvae, treated or untreated with Mtz.

To test whether Mtz-induced impairment of neurosecretory NPO cells affects the stress response, we treated *Tg(otpECR6-E1b:mmGFP)* and *Tg(otpECR6-E1b:nfsb-GFP)* larvae with E2 medium alone or E2 medium supplemented with Mtz and then subjected them to a mechanical stress paradigm that has been shown to induce an intensity-dependent increase in whole-body cortisol levels in zebrafish larvae (Castillo-Ramirez, L., unpublished data). Neither genotype nor Mtz treatment affected basal cortisol levels (Figure 7B; Two way ANOVA, genotype factor: F_(1,20)_ = 0.57, p = 0.46, Mtz factor: F_(1,20)_ = 2.69, p = 0.12, genotype X Mtz factor: F_(1,20)_ = 0.65, p = 0.43). Stressor exposure increased cortisol level in both *Tg(otpECR6-E1b:nfsb-GFP)* and *Tg(otpECR6-E1b:mmGFP)* larvae, regardless of Mtz incubation (Figure 7C; One sample t-test against 0, GFP –Mtz: t_(5)_ = 16.6, p < 0.0001, GFP + Mtz: t_(5)_ = 7.1, p = 0.0009, nfsb-GFP –Mtz: t_(5)_ = 18.4, p < 0.0001, nfsb-GFP + Mtz: t_(5)_ = 10.7, p < 0.0001). However, although Mtz treatment reduced stressor-induced cortisol increase in both groups, the reduction was much greater in *Tg(otpECR6-E1b:nfsb-GFP)* larvae (Figure 7C; Two way ANOVA, genotype factor: F_(1,20)_ = 8.90, p = 0.007, Mtz factor: F_(1,20)_ = 130.1, p < 0.0001, genotype X Mtz factor: F_(1,20)_ = 5.54, p = 0.03). In fact, cortisol increase differed between the two genotypes only in the presence of Mtz (Figure 7C; Bonferroni post hoc test, GFP –Mtz vs. nfsb-GFP –Mtz, p > 0.05; GFP + Mtz vs. nfsb-GFP + Mtz, p < 0.01). This provides evidence for the Mtz-induced impairment of the endocrine response in *Tg(otpECR6-E1b:nfsb-GFP)* larvae supporting the use of *otp*ECR6 for functional manipulation of the HPI axis.

## Discussion

Here, we report the identification and functional characterization of a *cis*-regulatory module (CRM) of the gene *Otp* with regulatory activity in the main stress-controlling hypothalamic region (NPO) in the zebrafish brain. This CRM is highly conserved in jawed vertebrates, and it restricts gene expression to a forebrain area which includes the neurosecretory cells involved in the HPI axis. Accordingly, when *otp*ECR6 was used to pharmacologically ablate these cells, we observed a significant impairment of the stress response.

Otp is an ancient transcription factor gene with a conserved expression in the vertebrate brain. Similar to other developmental regulators, the *Otp* gene is flanked by highly conserved non-coding sequences. Identifying precise functions of such sequences represents one of the pressing challenges of modern genomics research. Although evolutionary conservation at the sequence level indicates selective constraints against mutations [43], the correlation of ECRs with gene regulatory functions is not straightforward [44,45]. We used the zebrafish as a model organism to evaluate the *cis*-regulatory potential of a selected group of ECRs identified through extensive comparisons of the intergenic sequences of the *Otp* gene in representative species of vertebrates. We defined 6 ECRs consisting of a minimum of 80 bases with more than 70% nucleotide identity in the upstream region of the gene. Interestingly, none of the ECRs were evident in the genomes of the lampreys studied, although we found a copy of the *Otp* gene. This could be due to intrinsic problems in the genome assembly in these organisms due to the programmed genome rearrangement occurring in certain somatic tissues during embryonic development [46]. Alternatively, the ECRs could have evolved specifically in the ancestor of gnathostomes just after the second genome duplication event that occurred in the cyclostome-gnathostome lineage [47]. It is important to note that many neuropeptide genes whose identities are specified by Otp in gnathostomes are also present in cyclostomes [15], suggesting that an ancestral genetic network mediated by Otp in the diencephalon was already established in cyclostomes.

In our approach, we evaluated the regulatory potential of individual ECRs in zebrafish larvae. As a framework, we looked for GFP reporter activity of individual ECRs in the context of the Otp expression domain. Surprisingly, only one ECR was shown to be a true CRM, the element *otp*ECR6. The inability to detect GFP expression with the other ECRs points to a combinatorial use of these regions to achieve a regional activity [48]. Alternatively, some ECRs are used by repressors and will not activate the reporter. Moreover, the PCR product representing some of the tested ECRs could have omitted non-conserved critical transcription factor-binding sites required to maintain the full activity of the region. To prevent this problem, ECRs were flanked by more than 100 bp.

Interestingly, the identified CRM, *otp*ECR6, is included in the human enhancer hs262 reported in the Vista Enhancer Browser resource for conserved non-coding elements of the human genome and tested in mouse embryos (http://enhancer.lbl.gov). The enhancer hs262 displays a conspicuous lacZ activity in a restricted area of the mouse ventral forebrain, similar to the *otp*ECR6 activity reported here. In addition, *otp*ECR6 overlaps with a DNA track, reported in the Encyclopedia of DNA elements (ENCODE), showing significant levels of enrichment of the H3K27Ac histone mark, which is one of the characteristic signatures of an active enhancer (ENCODE Project Consortium, 2011). Together, these data suggest that *otp*ECR6 is an evolutionarily conserved DNA element with a conserved regulatory function in the forebrain of vertebrates.

Evolution of a gene’s function may occur through modifications of the architecture of its regulatory networks [49,50]. A whole-genome duplication event occurred in the ancestor lineage of ray-finned fishes approximately 350 million years ago [51]. This duplication event is proposed to explain the tremendous diversification of teleosts by releasing an important set of genomic mechanisms that allowed the evolution of gene function [52]. In zebrafish, the *otp* gene is present in two copies, *otpa* and *otpb*. We noticed that the percentage of identity of *otp*ECR6 was higher when comparing the *otpa* copy with the orthologous *Otp* genes than when comparing the same region of the *otpb* copy. In addition, the ECR landscape of the *otpb* locus lacks ECRs 3, 4 and 5. At the amino acid level, Otpa resembles more the mammalian counterpart [11]. In a recent paper, it has been shown that Otpa and Otpb have partially redundant roles in the development of the zebrafish diencephalon; however, Otpa has a more prominent role in the specification of neuroendocrine and dopaminergic cells [22]. Furthermore, the expression domains of *otpa* and *otpb* differ in the NPO and its surrounding areas in 5 dpf zebrafish larvae, where the *otpb* gene is expressed in a medial subregion within a larger *otpa* domain and *otpa* extends into the caudal telencephalon [28]. These results support the idea that the paralogous *otp* gene in zebrafish, *otpb*, has undergone a process of subfunctionalization through modifications at the *cis*-regulatory and amino acid levels [53].

The analysis of the chemoarchitecture defined by *otp*ECR6 identified neurosecretory cells involved in the HPI axis. For instance, *otp*ECR6 reporter activity colocalized with a group of cells expressing Crh, Avp, or Oxt in the neurosecretory preoptic area. Because Otp is required for the specification of neurosecretory lineages in zebrafish and mice, we can infer that the function of *otp*ECR6 is to maintain the regional expression of the *otp* gene in the NPO or in the homologous region of the mammalian hypothalamus, the PVN. In a recent screen to identify DA *cis*-regulatory modules in genes involved in DA neuron specification or function, Fujimoto et al. [30] reported a DNA element of the *otpb* gene, the otpb.A enhancer, which drove expression in the diencephalic DA cells and in the Oxt cells of the NPO. *otp*ECR6 is included within the 4.5 kb otpb.A enhancer. Interestingly, we did not observe any DA neurons colocalizing with GFP in *otpa*ECR6 stable lines. This suggests that the 4.5 kb otpb.A enhancer is in fact a composite regulatory module containing *otp*ECR6, which drives the expression in the NPO and unidentified regulatory sequences responsible for the expression in DA cells.

The restricted expression activity displayed by *otp*ECR6 in the caudal telencephalon and NPO area, and in particular in the neurosecretory cells involved in the HPI axis, may be useful for functional analyses of the stress response in vertebrates. The zebrafish is a promising model organism to study the mechanisms underlying early stress effects on the development of adult phenotypes [27]. Many of the cellular and molecular components of the stress axis are conserved between mammals and fishes [19,54], and the present work identified striking conservation at the gene regulatory level. We succeeded in altering the normal cortisol response to a mechanical stressor using conditional Mtz impairment in cells expressing the bacterial nitroreductase gene under the *otp*ECR6 regulatory element. These results were consistent with structural changes of nitroreductase-expressing cells when exposed to Mtz (e.g., reduced projections to the pituitary). We cannot exclude potential effects on the stress response from the uncharacterized cells in the caudal telencephalon or the few ectopic dorsal midbrain cells. The otpb.A enhancer was successfully used recently in a similar chemical ablation experiment to study the role of the diencephalic DA tract (DDT) in the locomotor development of zebrafish larvae [55]. Because *otp*ECR6 does not cover DA Otp+ cells, this regulatory element can be of use to control for any effect of non-DA Otp+ cells in locomotor and behavioral experiments.

New molecular tools for neuronal activity analysis (such as GCamPs), neuronal manipulation (such as Channelrhodopsins) or single-cell morphology visualization (Brainbow) can be coupled to this regulatory element to address questions concerning the differential activation of the HPI axis under acute and chronic stress.

Our results raise further questions: 1) Is *otp*ECR6 indispensable for the proper development of the neurosecretory hypothalamus in vertebrates? 2) If the function of *otp*ECR6 is conserved in humans, is there any genetic variation found in this element that is associated with any stress-related condition? 3) What type of regulatory mechanisms drive *otp*ECR6-dependent Otp expression? The answers to these questions promise to reveal new insights into the genetic networks regulating the stress axis.

## Conclusions

Using a combination of comparative genomics, zebrafish transgenesis and imaging, we found that the transcription factor Otp uses a single and highly conserved *cis*-regulatory element, *otp*ECR6, to achieve expression in a key neurosecretory control center in zebrafish. Our results suggest that the regulatory network used by the *Otp* gene in the hypothalamus was already established in the ancestor of gnathostomes. In addition, we showed that the response of the HPI axis to a mechanical stressor in zebrafish larvae is impaired when the function of the cells covered by the regulatory activity of *otp*ECR6 is compromised using a pharmacogenetic ablation technique. Consequently, we propose this element as a tool for further functional analyses of the physiology and ontogeny of the stress response in zebrafish.

## Methods

### Fish maintenance

Zebrafish were bred and maintained according to standard methods [56]. Zebrafish experimental procedures were performed according to the guidelines of the German animal welfare law and approved by the ethics committee of the local government (Regional Council Karlsruhe, Germany).

### PCR and plasmid generation

Individual identified evolutionarily conserved non-coding regions (ECRs) were PCR amplified from genomic DNA extracted from 3 dpf zebrafish larvae using the primers listed in Additional file 2: Table S1. PCR products were cloned into a modified version of the pZED vector [37]. The modification consisted of the removal of the Gateway cassette, replacement of the GATA basal promoter and the EGFP with the adenovirus E1B basal promoter and the use of mmGFP [57]. The test cassette was flanked by insulator elements that reduce positional effects [58] and the plasmid contained, in antisense, a cassette expressing RFP under the cardiac myosin light chain 2 promoter as a transgenesis marker.

In addition, 3x multimerized *otp*ECR6 was subcloned to generate different sets of plasmids: pT2-*otp*ECR6-E1B:RFP-CAAX, where RFP-CAAX denotes a membrane-bound red fluorescent protein and pT2-*otp*ECR6-E1B:*nfsb*-GFP, where *nfsb*-GFP encodes a chimeric protein consisting of the *E. coli* nitroreductase gene *nfsb1* N-terminally fused to GFP. In all cases, the identity of the cloned fragments was confirmed by sequencing.

### Plasmid injection and trangenesis

Recombinant plasmids were injected into one-cell stage wild-type embryos at a 10 ng/μl concentration in the presence of 10 ng/μl Tol2 transposase mRNA and 0.05% phenol red. The progenies of injected fish were maintained in E2 medium supplemented with 0.2 mM N-phenylthiourea (Sigma-Aldrich, catalogue no. P7629) to prevent pigmentation. Larvae were screened for heart RFP and brain GFP expression at 2, 3, or 5 dpf using a Leica MZ6 fluorescent dissecting microscope. At least 3 founders per construct were identified. The following transgenic lines have been established: *Tg(otpECR6-E1b:mmGFP)hd12, Tg(otpECR6-E1b:RFP-CAAX)hd13, Tg(otpECR6-E1b:nfsb-GFP)hd14.*

### Immunohistochemistry and fluorescent *in situ* hybridization

Immunohistochemistry on 3 dpf and 5 dpf larvae was performed as previously described [28] using the following antibodies. Primary antibodies: chicken anti-GFP (1:500, Abcam); rat anti-Otpa (1:500) [23]; rabbit anti-Avp (1:250) and rabbit anti-Oxt (1:250) [28]; rabbit anti-TH (1:250) [11]; rabbit anti-ACTH (1:1000, National Hormone and Peptide Program, National Institute of Diabetes and Digestive and Kidney Diseases); and rabbit anti-tagRFP (1:500, Evrogen). Secondary antibodies: goat anti-chicken Alexa Fluor 488 (1:1000, Invitrogen); goat anti-rabbit Alexa Fluor 555 (1:1000, Invitrogen); and goat anti-rat Alexa Fluor 555 (1:1000, Invitrogen).

Fluorescent *in situ* hybridization was performed as previously described [23] using the following *in situ* probes: *crh, avp, oxt, sst1.1,* and *penka,* as reported in [28]. Larvae were imaged in 80% glycerol in PBS using a Nikon 20x glycerol objective and a Leica SP5 confocal laser scanning microscope. Confocal image channels were recorded sequentially with optimal gain, offset, zoom, and dimension settings for the desired volume. Stacks were subsequently evaluated using Amira 5.4 software (FEI Visualization Sciences Group) to create maximum intensity projections, perform 3D registration, and reconstruct the spatial arrangement of cells [28]. Maximum intensity projections were restricted to the volume of interest, excluding signals from above or below. Brightness and contrast were adjusted for each channel.

### Conditional targeted cell-ablation

Conditional targeted cell-ablation was performed as reported [41] using a solution of 10 mM Mtz (Sigma-Aldrich catalog no. M1547) in E2 medium supplemented with 0.2 mM N-phenylthiourea (PTU). The experimental treatments were as follows: *nfsB*-GFP-positive or *nfsB*-GFP-negative embryos were sorted at 3 dpf and then transferred to either E2 + PTU media or E2 + PTU + Mtz media in 35-mm petri dishes. Embryos were maintained in the dark at 28°C for 48 h, and the medium was changed at 24 h. At 5 dpf, the embryos were transferred to E2 + PTU media for 24 h under normal 12 h light/12 h dark conditions, fixed in 4% paraformaldehyde for immunohistochemistry, and subjected to a TUNEL assay (In Situ Cell Death Detection Kit, TMR red, Roche). To examine the stress response, a pool of 30 embryos was used as an experimental unit, and 6 experimental units were tested per treatment. Mtz treatment was performed as above, but the E2 media were not supplemented with PTU.

### Stressor treatment and cortisol assay

Larvae were subjected to a stressor paradigm, which utilized hydrodynamic flow stimulation (HF) using a Variomag Poly 15 stirrer plate (Thermo Scientific) and a plastic-covered stirrer (Magnetic stir bar micro PTFE 6 mm × 3 mm; Fisher Scientific) (Castillo-Ramirez, L., unpublished data). The stimulation was performed for 3 minutes using a speed of 330 rpm. Samples were collected 10 minutes after onset of stimulation. For collection of samples, ice-cold water was added to the individual petri dishes to immobilize the larvae. They were then transferred to Eppendorf tubes, the remaining medium was removed, and the larvae were frozen in an ethanol/dry ice bath. The samples were stored at -20°C. All stimulations and collection of samples were performed between 11:00 and 14:00. The cortisol ELISA assay was performed as previously described [59].

### Statistical analysis

Cortisol data are shown as mean and standard error of the mean (S.E.M.). We used One sample t-tests (against 0 values) and Two way ANOVAs, followed by Bonferroni’s *post-hoc* tests for multiple group comparisons. Analyses were carried out using MS-Excel (Microsoft; Redmond, WA, USA) and Prism 5(Graphpad Software Inc.; San Diego, CA, USA).

## Additional files

Additional file 1: Figure S1. ClustalW 2.1 nucleotide alignment output of the individual ECRs flanking the *otp* gene in gnathostomes. The table below each alignment shows the length of the ECR identified in every species and the pairwise percentage of identity. The TATA box is underlined in the ECR1 alignment. Hs: *Homo sapiens*; Lc: *Latimeria chalumnae*; Dr: *Danio rerio*; Cm: *Challorhincus milii.*
Additional file 2: Table S1. Primer sequences used in the present work to amplify individual ECRs of the *otpa* gene.

## Abbreviations

Avp: Arginine vasopressin
ACTH: Adrenocorticotropic hormone
Cck: Cholecystokinin
Crh: Corticotropin-releasing hormone
CRM: *cis*-regulatory module
DA cells: Dopaminergic cells
ECR: Evolutionarily conserved region
FISH: Fluorescent *in situ* hybridization
GCs: Glucocorticoids
HPA: Hypothalamic-pituitary-adrenal axis
HPI: Hypothalamic-pituitary-interrenal axis
IHC: Immunohistochemistry
Mtz: Metronidazole
NM: Nitroreductase-metronidazole system
NPO: Neurosecretory preoptic area
Otp: Orthopedia transcription factor
Oxt: Oxytocin
Penka: Proenkephalin a
Sst1.1: Somatostatin
Vip: Vasoactive intestinal peptide
