# Supplementary material for: A vertebrate-conserved cis-regulatory module for targeted expression in the main hypothalamic regulatory region for the stress response
Source: BMC Dev Biol. 2014 Nov 27;14:41. doi: 10.1186/s12861-014-0041-x (PMC4248439; doi:10.1186/s12861-014-0041-x)
Supplement: Additional file 1: Figure S1. — ClustalW 2.1 nucleotide alignment output of the individual ECRs flanking the otp gene in gnathostomes. The table below each alignment shows the length of the ECR identified in every species and the pairwise percentage of identity. The TATA box is underlined in the ECR1 alignment. Hs: Homo sapiens; Lc: Latimeria chalumnae; Dr: Danio rerio; Cm: Challorhincus milii. [file 12861_2014_41_MOESM1_ESM.pdf]

A) <u>**ECR1**</u>

```
Hs-ECR1          CTGACATTTGCAGGCTGCCCTCCTGATTGGTCCCCTCGCCGAGCTGCTCACGGGTTCATT
Cm-ECR1          CTGACATTTGCAGCGTTGGGTTTTGATTGGCCCTGATTTGGAGCTGCTCATGGGCTCATT
Lc-ECR1          CTGACATTTGCAGCCTTCCAATCTGATTGGTCCTCATTTGGAGCAGCTCATGGGTTCATT
Dr-ECR1          CTGACACCTCCAGGCTTCAACGCTGATTGGCTCTTATTTGAAGGAGCTCATGGGTTCATT
                 ******  * ***  *       *******  *        **  ***** *** *****

Hs-ECR1          CAACTGTTAAGCACCTCCCCGTCTCTCTAAAGGACATTATAATGCAAGAAGCCCCCTTTT
Cm-ECR1          CAACTGTTAAGCACCACCCCGTCCCTCTAAAGGAGATTATAATGCAGGAGACCCCCTTTT
Lc-ECR1          CAACTGTTAAGCACCTCCCAGTCTCTCTAAAGGACATTATAATGCAAGAGACCCCCTTTT
Dr-ECR1          CAACTATTAAGCGCCTCCCAGTTCCTCTAAAGGACATTATAATGCAAGGAACCTCCTTTT
                 ***** ****** ** *** **  ********** *********** *   ** ******

Hs-ECR1          TAACCACAAACCGAA
Cm-ECR1          --ACAACAAACAGAA
Lc-ECR1          TAACCACAAACCGAA
Dr-ECR1          TAACCACAAACCGAA
                   ** ****** ***
```

Sequence 1: Hs-ECR1	135 bp	Sequences (1:2) Aligned. Score:	88
Sequence 2: Lc-ECR1	135 bp	Sequences (1:3) Aligned. Score:	78
Sequence 3: Dr-ECR1	135 bp	Sequences (1:4) Aligned. Score:	80
Sequence 4: Cm-ECR1	133 bp	Sequences (2:3) Aligned. Score:	85
		Sequences (2:4) Aligned. Score:	87
		Sequences (3:4) Aligned. Score:	78

B) <u>**ECR2**</u>

```
Hs-ECR2          CAGCCAAGTCTAAAC-AGTAGTAAATTAGTACAAATTTATACTGATTTTACTCTAATAAT
Cm-ECR2          CAGCCAAGTCTAAACCAGGAGTAAATTAGTACACATTTATACTGATTTTATTTTAATAAT
Lc-ECR2          CAGCCAAGTCTAAAC-ACTAGTAAATTAGTACAAATTTATACTGATTTTACTCTAATAAT
Dr-ECR2          CAGTCAGGCCTAAAC-ACTACTAAATCAGCCCTAATTTATACTGATCTCGC--TAATAAT
                 *** ** * ****** *  * ***** **  *  ************ *     *******

Hs-ECR2          CGTAATAAAA-GCTTATAGATTTGGCACTAATTACATAAATGCCTAATGATCTTGAAAAT
Cm-ECR2          CGTAATAAAA-GCTTATAGATTTGGCACTAATTAGATAAATGCCTAATGATCTTGAAAAT
Lc-ECR2          CGTAATAAAA-GCTTATAGATTTGGCACTAATTACATAAATGCCTAATGATCTTGAAAAT
Dr-ECR2          CGTAATAAAAAGCTTATAGATTTGGCACTAATTACATAAAAGCCTAATGATGTTGAAAAT
                 ********** *********************** ***** ********** ********
```

Sequence 1: Hs-ECR2	118 bp	Sequences (1:2) Aligned. Score:	99
Sequence 2: Lc-ECR2	118 bp	Sequences (1:3) Aligned. Score:	88
Sequence 3: Dr-ECR2	117 bp	Sequences (1:4) Aligned. Score:	95
Sequence 4: Cm-ECR2	119 bp	Sequences (2:3) Aligned. Score:	88
		Sequences (2:4) Aligned. Score:	94
		Sequences (3:4) Aligned. Score:	83

C) <u>**ECR3**</u>

```
Hs-ECR3          TGTTTATACTGCTATAAAAATCAAGACCAATAGAAAAGTCATAAAATGAAGCGGGCTATC
Lc-ECR3          TGTTTATACTGCTATAAAAATCAAGAGCAATAGAAAAGTCATAAAATGAAGAGCGCTATA
Dr-ECR3          TGTTTGTACAGCTATAAAAGGATCGAGCTATTGAAAAGTCGTAAAATGAAGCGCGCTATC
                 ***** *** *********     ** * ** ******** ********** * *****

Hs-ECR3          AATCATGCCGCAACATTGTT
Lc-ECR3          AATCAGCCAGCAATCTCGCT
Dr-ECR3          AATCACGCTGTAACCGTGTT
                 *****  * * **    * *
```

Sequence 1: Hs-ECR3	80 bp	Sequences (1:2) Aligned. Score:	82
Sequence 2: Lc-ECR3	80 bp	Sequences (1:3) Aligned. Score:	78
Sequence 3: Dr-ECR3	80 bp	Sequences (2:3) Aligned. Score:	72

D) **ECR4**

```
Hs-ECR4          CAGAATTACATTTGTTTTTCTTGTGATTTTATTAAAAGTCACTCCTCATCTCCAGAATGC
Lc-ECR4          CAGAATTACATTTGTTTTTCTTGTGATTTTATTAAAAGTCACTCCTCATCTCCAGAATGC
Cm-ECR4          TAGAATTACATTTGTTTT-CTTGTGATATTATTAAAAGTCTATCCTCATCTCCAGAGTGC
Dr-ECR4          CAGAATTACATTTGTTTTTCTTGTGATTTTATTAAAAGTCACTCCTCATCTCCAGAGCGC
                  ***************** ******** ************  **************  **

Hs-ECR4          GTGAAAATATCTACTTTCCACTCAGATACACCGCATTAACTTGTTGGAGGCGAATTTGTT
Lc-ECR4          GTGAAAATATCTACTTTCCACTCAGATACACCACATTAACTTGTTGGAGGCGAATTTGTT
Cm-ECR4          GTGAAAATATCTACTTTCCACTCAGATACACCGCATTAACTTGTTCGAGGCGAATTTGTT
Dr-ECR4          GTGAAAATGTCTACTTTCCACTCAGATGCGCCGCATTAACTTGCAGGGCGCGAGTTTGTT
                 ******** ****************** * ** **********   *  **** ******

Hs-ECR4          TGTTTGTCTATTTT-ATTTATTTGCTAGAT---AAGATTGATGCAGTCTTATTAGC
Lc-ECR4          TGT----CTATTTT-ATTTATTTGCTGGAT---AAGATTGATGCAGTCTTATTAGC
Cm-ECR4          TGTTTGTCTATTTTTATTTATTTGGGGGAGGCGAAGATTGATGTGTTGTTATTATC
Dr-ECR4          TGTTTGTCTATTTTTATTTATATGCAGTGT---AAGATTGATGCGAGCTTGTTATC
                 ***    ******* ****** **         **********     ** *** *
```

Sequence 1: Hs-ECR4	172 bp	Sequences (1:2) Aligned. Score:	98
Sequence 2: Lc-ECR4	168 bp	Sequences (1:3) Aligned. Score:	88
Sequence 3: Dr-ECR4	173 bp	Sequences (1:4) Aligned. Score:	90
Sequence 4: Cm-ECR4	175 bp	Sequences (2:3) Aligned. Score:	88
		Sequences (2:4) Aligned. Score:	90
		Sequences (3:4) Aligned. Score:	85

E) **ECR5**

```
Hs-ECR5          CTGTCACCAAATCACCTGCACGAAACAAGAATCTAATTTGTTAAGCTGGCATGTCTGCAG
Lc-ECR5          CTGTCACCAAATCACCTGCACGAAACAAGAATCTAATTTGTTAAGCTGGCATTTCTGCAG
Cm-ECR5          CTCTCACAAAATCACCTGCACGAAACAAGAATCTAATTTGTTAACCTGGCATTTCTGTAG
Dr-ECR5          CTGTCACCGCATCACCTGCGCGAAACAAGAATCTAATTTGTTAAACTGGCATTTCTGCAG
                 ** ****   ********* ************************ ******* **** **

Hs-ECR5          ATGGAAGATCGATCTTCTCTGTAGATTTCTCTAATTGAGTGAATATAGACGCCC
Lc-ECR5          ATGGAAGATCGATTTTCTCTTTAGATTTCTCTAATTGAGTGAATATAGACGCCC
Cm-ECR5          ATGGAAGATCGATTTTCTCTTTAGAATTCTCTAATTGAGTGAATATAGACGCCC
Dr-ECR5          ATGGACGATCGATTTTCTCTTTAGAATTCTCTAATTGAGTGAATATAGACGCCC
                 ***** ******* ****** **** ****************************
```

Sequence 1: Hs-ECR5	114 bp	Sequences (1:2) Aligned. Score:	97
Sequence 2: Lc-ECR5	114 bp	Sequences (1:3) Aligned. Score:	92
Sequence 3: Dr-ECR5	114 bp	Sequences (1:4) Aligned. Score:	92
Sequence 4: Cm-ECR5	114 bp	Sequences (2:3) Aligned. Score:	94
		Sequences (2:4) Aligned. Score:	95
		Sequences (3:4) Aligned. Score:	92

6) **ECR6**

```
Hs-ECR6          AATGAGACCCCTTTACTCCGTGACGTGCAGCCGCTCCATCTCATTAAGGAAATCGCTCTT
Lc-ECR6          AACGAGACCCCTTTACTCCGTGACGTGCAACCGCTCCATCTTATTAAGGAAATCGCTCTT
Cm-ECR6          AATGAGACCCCCGGACTCCGTGACATGCAGCCGCTCCATCTCATTAATGAAATCGCTCTT
Dr-ECR6          AATGAGGCGCCGCCACTCCGTGACACCCAACCGCTCCATCTCGTTAAGGAAATCGCTCTT
Dr-ECR6b         AATGGAGGCCAGCTACTCCGTGACTTGCCAAAGCTCCATTTCATTACGGTAATCGCCGTC
                 ** *     *    **********   *    ******* *  ***  * ******  *

Hs-ECR6          CAATGCTGATTATTTTATTTGCAGCCATAATTATATTTCTTTCGGCTAAATCACGGTTTA
Lc-ECR6          CAATGCTGATTATTTTATTTGCAGCTATAATTATATTTCTTTCGGCTAAATCACGGTTTA
Cm-ECR6          CAATGCTGATTATTTTATTTGGAACCATAATTATATTTCTTTTGGCTGAATCACGATTTA
Dr-ECR6          CAATGCTGATTATTTTATTTGGAGCCATAATTATATTTCTTTCGGATAAATCACGGTCTA
Dr-ECR6b         CATTGCTGATTATTTTATTTAG-GCCATAATTGTATTCTCCCCAGATAACTCACGTTGAA
                 ** *****************    * ****** ****       * * * ***** *  *
```

```
Hs-ECR6           ATCGAGCCCACAT--GGAGGGCAGCAGCACTAAT
Lc-ECR6           ATCGAGCCCACAT--GGAGGGCAACTGGTTTAAT
Cm-ECR6           ATAGAGCACACGTTCGGGGGCAAAGCGCTGTAAT
Dr-ECR6           ATCGAGTCCGTTC--GGAGGGCAGCGCCTCTAAT
Dr-ECR6b          ATCGGGTCCACAT--GGAGGGCACCAAGTCTAAT
                  ** * *  *      ** **  *       ****
```

Sequence 1: Hs-ECR6 152 bp<br>Sequence 2: Lc-ECR6 152 bp<br>Sequence 3: Dr-ECR6 152 bp<br>Sequence 4: Dr-ECR6b 151 bp<br>Sequence 5: Cm-ECR6 154 bp	Sequences (1:2) Aligned. Score: 94<br>Sequences (1:3) Aligned. Score: 86<br>Sequences (1:4) Aligned. Score: 70<br>Sequences (1:5) Aligned. Score: 84<br>Sequences (2:3) Aligned. Score: 83<br>Sequences (2:4) Aligned. Score: 69<br>Sequences (2:5) Aligned. Score: 80<br>Sequences (3:4) Aligned. Score: 72<br>Sequences (3:5) Aligned. Score: 84<br>Sequences (4:5) Aligned. Score: 59
