# Supplementary figures and images for: A vertebrate-conserved cis-regulatory module for targeted expression in the main hypothalamic regulatory region for the stress response
Source: BMC Dev Biol. 2014 Nov 27;14:41. doi: 10.1186/s12861-014-0041-x (PMC4248439; doi:10.1186/s12861-014-0041-x)

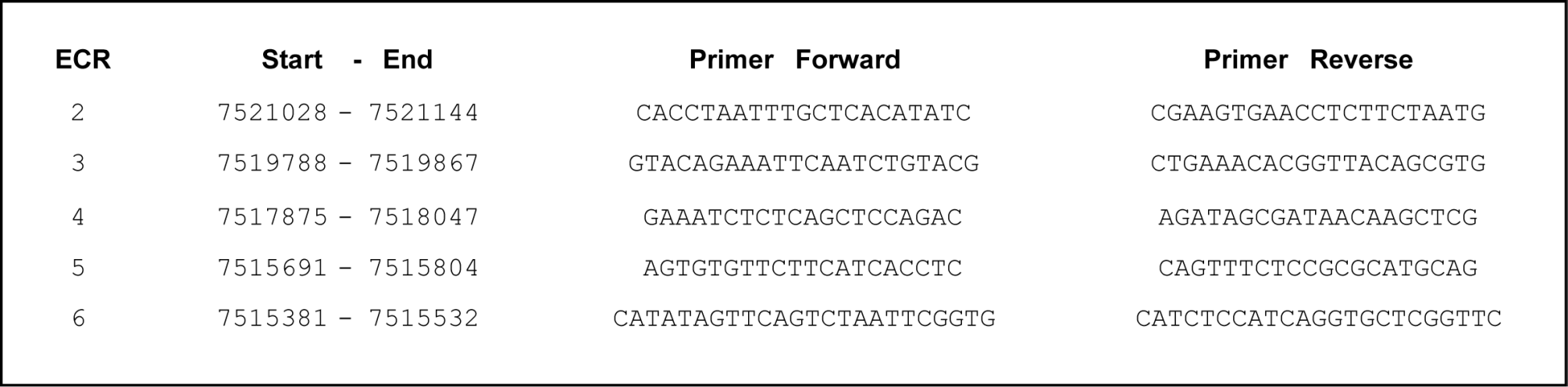

Supplement: Additional file 2: Table S1. — Primer sequences used in the present work to amplify individual ECRs of the otpa gene. [file 12861_2014_41_MOESM2_ESM.tiff]
